# Discovery of genetic variants of the kinases that activate tenofovir among individuals in the United States, Thailand, and South Africa: HPTN067

**DOI:** 10.1371/journal.pone.0195764

**Published:** 2018-04-11

**Authors:** Dominique B. Figueroa, Joseph Tillotson, Maoji Li, Estelle Piwowar-Manning, Craig W. Hendrix, Timothy H. Holtz, Kevin Bokoch, Linda-Gail Bekker, Frits van Griensven, Sharon Mannheimer, James P. Hughes, Robert M. Grant, Namandjé N. Bumpus

**Affiliations:** 1 Department of Pharmacology & Molecular Sciences, Johns Hopkins University School of Medicine, Baltimore, MD, United States of America; 2 Department of Medicine (Division of Clinical Pharmacology), Johns Hopkins University School of Medicine, Baltimore, MD, United States of America; 3 Vaccine and Infectious Disease Division, Fred Hutchinson Cancer Research Center, Seattle, WA, United States of America; 4 Department of Pathology, Johns Hopkins University School of Medicine, Baltimore, MD, United States of America; 5 HIV/STD Research Program, Thailand Ministry of Public Health–U.S. Centers for Disease Control and Prevention Collaboration, Nonthaburi, Thailand. Division of HIV/AIDS Prevention, U.S. Centers for Disease Control and Prevention, Atlanta, Georgia, United States of America; 6 FHI 360, Research Triangle Park, NC, United States of America; 7 The Desmond Tutu HIV Centre, University of Cape Town, Cape Town, South Africa; 8 Department of Epidemiology and Biostatistics, University of California, San Francisco School of Medicine, San Francisco, CA, United States of America; 9 Department of Epidemiology and Department of Medicine, Columbia University Mailman School of Public Health, New York, NY, United States of America; 10 Department of Biostatistics, University of Washington, Seattle, WA, United States of America; 11 Gladstone Institutes, University of California, San Francisco, San Francisco, CA, United States of America; Virginia Commonwealth University, UNITED STATES

## Abstract

Tenofovir (TFV), a nucleotide reverse transcriptase inhibitor, requires two phosphorylation steps to form a competitive inhibitor of HIV reverse transcriptase. Adenylate kinase 2 (*AK2*) has been previously demonstrated to phosphorylate tenofovir to tenofovir-monophosphate, while creatine kinase, muscle (*CKM*), pyruvate kinase, muscle (*PKM*) and pyruvate kinase, liver and red blood cell (*PKLR*) each have been found to phosphorylate tenofovir-monophosphate to the pharmacologically active tenofovir-diphosphate. In the present study, genomic DNA isolated from dried blood spots collected from 505 participants from Bangkok, Thailand; Cape Town, South Africa; and New York City, USA were examined for variants in *AK2*, *CKM*, *PKM*, and *PKLR* using next-generation sequencing. The bioinformatics tools SIFT and PolyPhen predicted that 19 of the 505 individuals (3.7% frequency) carried variants in at least one kinase that would result in a decrease or loss of enzymatic activity. To functionally test these predictions, AK2 and AK2 variants were expressed in and purified from *E*. *coli*, followed by investigation of their activities towards tenofovir. Interestingly, we found that purified AK2 had the ability to phosphorylate tenofovir-monophosphate to tenofovir-diphosphate in addition to phosphorylating tenofovir to tenofovir-monophosphate. Further, four of the six AK2 variants predicted to result in a loss or decrease of enzyme function exhibited a ≥30% decrease in activity towards tenofovir in our in vitro assays. Of note, an AK2 K28R variant resulted in a 72% and 81% decrease in the formation of tenofovir-monophosphate and tenofovir-diphosphate, respectively. These data suggest that there are naturally occurring genetic variants that could potentially impact TFV activation.

## Introduction

Tenofovir (TFV) is a nucleotide reverse transcriptase inhibitor that, when administered as the prodrug tenofovir disoproxil fumarate (TDF), is used for the treatment and prevention of human immunodeficiency virus (HIV) infection [1]. In fact, co-formulated emtricitabine/tenofovir disoproxil fumarate is currently the only drug approved by the FDA for use in HIV pre-exposure prophylaxis (PrEP) [2], although the CDC and WHO recommend TDF alone as an alternate PrEP regimen. PrEP is a pharmacological strategy for preventing HIV infection, the efficacy of which has been bolstered by several clinical trials [3] including the iPrEX study comprised of 2,499 men who have sex with men (MSM) or transgender women (TGW) [4], the Botswana TDF2 study comprised of 1,219 men and women [5], and the Partners PrEP study, comprised of 4,747 serodiscordant couples [6]. The iPrEX study demonstrated a 92% reduction in HIV incidence among MSM and TGW who had any detectable PrEP medications in blood [4], and no participant became infected if drug levels indicated use of 4 or more tablets per week [7]. In the Botswana TDF2 study, 78% of HIV-uninfected men and women were protected against HIV when adjusted for adherence [5]. Finally, in the largest of these studies, Partners PrEP, a 90% decrease in HIV infection among HIV-uninfected partners in comparison to placebo was observed [6].

In order to produce the active HIV reverse transcriptase inhibitor, TFV must be phosphorylated twice, first to tenofovir-monophosphate (TFV-MP) [8]. TFV-MP then is phosphorylated to tenofovir-diphosphate (TFV-DP), which is the pharmacologically active form of TFV. Our lab has investigated this phosphorylation pathway and demonstrated the compartment specificity of the kinases involved in TFV activation [9]. Specifically, we found that adenylate kinase 2 (AK2) phosphorylates TFV to TFV-MP in peripheral blood mononuclear cells (PBMC), and in vaginal and colorectal tissue. Interestingly, while we observed phosphorylation of TFV-MP to TFV-DP by creatine kinase, muscle (CKM) in colorectal tissue, the kinases pyruvate kinase, muscle (PKM) and pyruvate kinase, liver and red blood cell (PKLR) catalyzed this step in PBMC and vaginal tissue [9]. Through this work, we proposed the possibility that genetic variants of these kinases could impact the kinase function and therefore TFV phosphorylation.

Adenylate kinases (AK) play a vital role in cellular energy metabolism and homeostasis. These enzymes maintain adenine nucleotide ratios by catalyzing the reversible phosphotransfer from ATP to AMP, resulting in two ADP molecules [10]. AK deficiency or mutations that compromise AK activity, like the nucleotide kinase-dead mutant (AK2 K28E), can lead to congenital disorders such as reticular dysgenesis and disruptions in normal cellular function [11, 12]. Like AK, creatine kinases also play a role in cellular energy homeostasis, specifically in tissue with high-energy demand such as striated muscles. Creatine kinases do this by utilizing ATP to convert creatine (Cr) to phosphocreatine (PCr), which serves as an energy pool for rapid regeneration of ATP [13]. Mutations in the CKM coding region compromise its activity. A D54G variant of CKM was unable to properly fold, resulting in decreased activity as well as its susceptibility to aggregation [14]. Pyruvate kinase catalyzes the rate-limiting step in glycolysis by converting phosphoenolpyruvate (PEP) to pyruvate and transferring the phosphate group to ADP, yielding one molecule of ATP [15]. Overexpression of PKs, specifically PKM2, have been seen in cancer cells, while deficiency in PKs have been linked to hemolytic anemias [15, 16]. Because it has been demonstrated that genetic mutations exist that can have deleterious effects on the activities of these kinases toward their naturally occurring substrates, we hypothesize that genetic variants of these kinases could also impact their ability to catalyze the phosphorylation of TFV.

Thus, in the present study, we analyzed genomic DNA isolated from dried blood spots (DBS) in 505 clinical trial participants across three geographic locations, namely Bangkok, Thailand (n = 171), New York City, USA (n = 149), and Cape Town, South Africa (n = 185). Of particular note, this study represents the first targeted resequencing analysis of the *AK2*, *CKM*, *PKM*, and *PKLR* genes in subjects in Asia. We have also expressed and purified 12 variants of AK2 to demonstrate the impact of these genetic variants on TFV phosphorylation. Taken together, this work establishes the concept that due to genetic variations, certain individuals may exhibit a decreased ability to activate TFV.

## Materials and methods

### Clinical study sites and sample collection

DBS were obtained from HIV-uninfected MSM or TGW and women who have sex with men (WSM) (n = 505) enrolled in the HIV Prevention Trials Network study HPTN 067. The study was conducted following local IRB approval at three clinical research sites (CRS): Emavudleni CRS in Cape Town, South Africa; Silom Community Clinic CRS in Bangkok, Thailand; and the Harlem Prevention Center CRS in New York, United States. Only subjects who provided written consent to genetic testing were genotyped. Each participant contributed five DBS containing approximately 50 μL of whole blood spotted onto Whatman^TM^ 903 Protein Saver Cards. Information about participants enrolled and genotyped in this study is summarized in Table 1.

**Table 1:** HPTN 067 participant enrollment location and gender information.

HPTN 067 study locations and participant demographics n = 505		
Study site	Reported gender	n (% of n)
Bangkok, Thailand	MSM, TGW	171 (34)
New York City, USA	MSM, TGW	149 (30)
Cape Town, South Africa	WSM	185 (36)

Study participants were enrolled across three geographic locations, with MSM and TGW enrolled in Bangkok (n = 171) and New York City (n = 149) and WSM enrolled in Cape Town (n = 185).

### Genomic DNA isolation from HPTN 067 DBS samples

Genomic DNA was isolated from two DBS from each individual using a QIAamp 96 DNA Blood Kit (QIAGEN, Valencia, CA). DBS were punched out using a hole puncher and the genomic DNA was extracted simultaneously from each of the two DBS (such that the extracted genomic DNA from the two DBS was combined) following the supplementary protocol “Isolation of genomic DNA from dried blood spots using the QIAamp ®96 DNA Blood Kit–(EN)” from QIAGEN. Purified DNA was eluted using 150 μL DEPC-treated, nuclease-free water (Quality Biological, Inc., Gaithersburg, MD) and concentrated using a ZR-96 DNA Clean-up Kit^TM^ (Zymo Research, Irvine, CA). The resulting concentrated and purified genomic DNA was eluted in 12 μL DEPC-treated, nuclease-free water.

### Next-generation sequencing target design, sample preparation, and analysis

Sequencing of the kinases *AK2*, *CKM*, *PKM* and *PKLR* was executed in each gene’s exonic regions using the Illumina TruSeq Custom Amplicon kit v1.5 (Illumina, San Diego, CA). Probes for sequencing the combination of these kinases were designed using Illumina DesignStudio software as previously described [9]. Genomic DNA isolated from clinical samples was processed following the Illumina TruSeq Custom Amplicon Library Preparation Guide (Part Number 15027983 Rev. C, August 2013). DNA concentration was measured using a Qubit® 3.0 Fluorimeter (Thermo Scientific, New York, NY). Fifty ng of DNA input were used per DNA sample sequenced. The resulting prepared DNA library (6 μL) was diluted in 594 μL HT1 buffer containing 1% PhiX sequencing control. Illumina VariantStudio software was used to annotate and analyze variant read quality as previously described [9]. To predict the consequences of the detected variants on kinase activity the in silico tools SIFT (sorts intolerant from tolerant substitutions; J. Craig Venter Institute online tool) and PolyPhen (polymorphism phenotyping; Harvard University online tool) were used. A SIFT score < 0.05 is commensurate with a prediction that the amino acid substitution is possibly deleterious and > 0.05 indicates a tolerated amino acid substitution. A PolyPhen score > 0.908 is suggestive of a probably damaging, 0.447–0.908 a possibly damaging, or < 0.447 a benign amino acid substitution [17, 18].

### Expression and purification of wild-type (WT) AK2

*E*. *Coli* BL21(DE3) cells (Agilent Technologies) were transformed with pET30a-AK2 plasmid (AK2 was purchased from Origene), grown in Luria Broth (LB) medium containing 50 μg/mL kanamycin at 37°C to an OD_600_ of 0.8. Cells were induced with 1 mM isopropyl β-D-1-thiogalactopyranoside (IPTG) and grown for an additional 3 h at 37°C. Cells were recovered by centrifugation and resuspended in lysis buffer (50 mM HEPES pH 7.3, 150 mM KCl, 10 mM MgCl_2_, 5% glycerol, 2 mM 2-mercaptoethanol, and 1x EDTA-free protease inhibitor cocktail (Roche)). Cells were lysed by sonication and lysate was clarified by centrifugation (15,000 rpm, 45 min, at 4°C). The resulting supernatant was applied to 1 mL of Superflow Co^2+^ resin (HiTrap TALON, GE Healthcare), washed with 10 column volumes of lysis buffer, and eluted using a gradient elution with lysis buffer supplemented with 500 mM imidazole. Following purification, AK2 protein was dialyzed in storage buffer (20 mM HEPES pH 7.3, 150 mM KCl, 10 mM MgCl_2_, 5% glycerol, and 2 mM 2-mercaptoethanol) and stored at -80°C.

### Expression and purification of AK2 variants

Site directed mutagenesis was performed using wild-type pET30a-AK2 as a template. Amino acid residues in AK2 were mutated using a modified quick-change procedure [19] to produce each of the 12 previously unreported variants of this kinase that were detected in this study (A8V, V19G, K28R, A52T, A55V, E59K, K62E, E77K, E155K, T194I, I206F, I206V). These experiments were performed using a QuikChange Lightning Kit from Agilent Technologies. All mutations were confirmed by DNA sequencing (The Synthesis and Sequencing Facility, John Hopkins University). Expression and purification of these variants were carried out in the same way as wild-type AK2, as described in the prior paragraph.

### AK2 activity assay, sample preparation, and detection of TFV-MP and TFV-DP formation

In a reaction volume of 200 μL, 500 nM recombinant proteins (wild-type AK2 or AK2 variants), 1 mM TFV, and 1 mM ATP were incubated in assay buffer (20 mM HEPES pH 7.3, 100 mM KCl, 10 mM MgCl_2_, 2 mM DTT) at 37°C for 2.5 h. A saturating concentration of TFV was used to ensure that substrate depletion would not underlie any differences observed in the formation of phosphates in these activity assays. A 100 μL aliquot was taken from the reaction, quenched in 1 mL methanol/formic acid (0.1%) solution, and incubated on ice for 10 min. Samples were then centrifuged for 10 min at 10,000 g and supernatant was dried under vacuum. Analysis of TFV, TFV-MP, and TFV-DP was done using a Dionex Ultimate 3000 uHPLC system coupled to a TSQ Vantage Triple Stage Quadrupole mass spectrometer (ThermoFisher Scientific). Separation of these analytes was done using a HALO C18, 100x2.1 mm, 2.7 μm particle size column (Advanced Materials Technology) at a flow rate of 0.45 mL/min. Mobile phase A (MPA) was 5 mM N,N-Dimethylhexylamine (DMHA) in water, pH 7.0, and B (MPB) was 5 mM DMHA in acetonitrile/water (50:50, v/v). A gradient program was used and is as follows: 0–53% MPB from 0–6 min, 53–100% MPB from 6–6.5 min, 100% MPB from 6.5–10 min, 100–0% MPB from 10–10.5 min, and 0% MPB from 10.5–20 min. Samples were reconstituted with 30 μL MPA, and injection volume was 1 μL. The ESI source of the TSQ Vantage mass spectrometer was operated in the positive ion mode and fragment ions were detected using the following transitions: *m/z* 288→176, 368→270, 488→270 for TFV, TFV-MP, and TFV-DP, respectively. Data were acquired and processed using Xcalibur (ThermoFisher Scientific).

### Statistical analysis

Differences of TFV-MP and TFV-DP formation between wild-type AK2 and AK2 variants were compared using a two-tailed unpaired *t* test. This was performed using GraphPad Prism (San Diego, CA), and significance was set as follows: *, p≤0.05; **, p≤0.01; ***, p≤0.001.

## Results

### Analysis of individuals carrying genetic variants in tenofovir-activating kinases *AK2*, *CKM*, *PKM*, and *PKLR*

**Fig 1:**
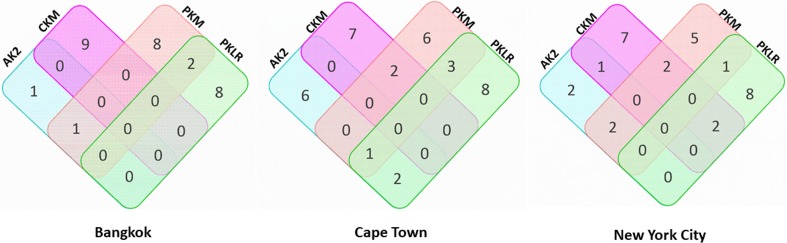
Distribution of individuals enrolled at the Bangkok, Cape Town, and New York City study sites carrying genetic variants in TFV-activating kinases. Each rectangle is representative of a TFV-activating kinase that was sequenced: *AK2* in blue, *CKM* in pink, *PKM* in orange and *PKLR* in green. Numerical values indicate the number of individuals detected to carry a single nucleotide variation or deletion. Overlapping regions of each rectangle indicate the number of individuals with genetic variants in more than one kinase.

Variant distribution among Bangkok study participants is demonstrated using a Venn diagram in Fig 1. At the Bangkok study site, 29 of the 171 participants sequenced (17%) for these kinases carried single nucleotide variants (SNVs) or deletions that were predicted to result in a mutation at the amino acid level. Of these 29 participants from the Bangkok study site, 26 individuals exhibited variants for a singular nucleotide kinase. Moreover, one individual had a variant detectable for only *AK2*, nine individuals had variants detectable for only *CKM*, eight individuals had variants detectable for only *PKM*, and eight individuals had variants detectable for only *PKLR*. Of the remaining three participants, one carried variants detectable in *AK2* and *PKM* and two had genetic variants in both *PKM* and *PKLR*.

At the New York City study site, 35 of the 149 participants sequenced (23%) were observed to carry SNVs or deletions predicted to result in a mutation at the amino acid level. The observed variant distribution among New York City study participants is depicted using a Venn diagram in Fig 1. Of the 35 participants from the New York City study site detected to carry a genetic variant, 27 exhibited variants for only one of the kinases sequenced. Six individuals carried variants for *AK2* alone, seven individuals for *CKM* alone, six individuals for *PKM* alone, and eight individuals carried variants for *PKLR* alone. Further, of these 35 participants, eight carried genetic variants in more than one kinase. One individual had detectable variants in *AK2*, *PKM*, and *PKLR*, two individuals were observed to have detectable variants in *AK2* and *PKLR*, two individuals were observed to have detectable variants in *CKM* and *PKM* and three individuals were observed to carry variants in *PKM* and *PKLR*.

Variant distribution among Cape Town study participants is shown using a Venn diagram in Fig 1. Thirty of the 185 participants (16%, 30/185) enrolled at this study site were observed to have SNVs or deletions predicted to result in a mutation at the amino acid level. Twenty-two of the 30 individuals carrying variants in Cape Town exhibited single nucleotide variants for only one of the kinases sequenced. Of these 22 individuals, two displayed variants in the kinase *AK2* alone, seven in the kinase *CKM* alone, five in the kinase *PKM* alone, and eight in the kinase *PKLR* alone. In contrast to exhibiting variants in only one kinase, eight participants carried genetic variants in more than one kinase. Two individuals were observed to have detectable variants in *AK2* and *PKM*. One individual was observed to have detectable variants in both *AK2* and *CKM*. Seven individuals were observed to have detectable variants in only *CKM*, while two were observed to have detectable variants in *CKM* and *PKM* and two to have detectable variants in *CKM* and *PKLR*. Five were observed to have detectable variants in *PKM*. Eight individuals were observed to have detectable variants in *PKLR*. One individual was observed to carry detectable variants in *PKM* and *PKLR*.

### *AK2* genetic variants detected across three geographic locations

Our laboratory has previously demonstrated *AK2* phosphorylation of TFV to TFV-MP in PBMC, vaginal tissue, and colon tissue [9]. This previous study identified 12 heterozygous missense variants that had never before been reported; however, none of the *AK2* variants identified through this previous work were found in our current study. In the 505 participants sequenced in this study, we observed 17 single nucleotide variations (SNVs) predicted to result in a mutation of the amino acid sequence for the DNA reference sequence corresponding to *AK2* (NM_001625.3). These specific variants were detected across 16 individuals. The described variants, which include both previously reported and novel variants, are detailed in Table A in S1 File. All 16 individuals were observed to be heterozygous for the variants detected. Further, of the 16 individuals observed to carry at least one SNV, two individuals were from the Bangkok study site, five individuals were of the Cape Town study site, and nine individuals were of the New York City study site. Among the individuals carrying at least one SNV, one individual at the Cape Town study site was observed to be carrying two variants while two individuals at the New York City study site were observed to be carrying two variants. Of the 17 *AK2* genetic variants observed, 13 of these variants, which were detected across 11 participants, were previously unreported (Table 2). Twelve of the 13-abovementioned variants were missense substitutions. Of the 505 individuals sequenced for *AK2* missense variants, one individual in New York City was observed to have the previously unreported variant at the coding DNA position c.664G>A. Applying *in silico* functional prediction tools to the variant c.664G>A, it is predicted to be “deleterious” and “probably damaging”. *In silico* tools SIFT and PolyPhen were used to predict the functional consequences of these variants as previously described [9]. Four variants contained in the Single Nucleotide Polymorphism database (dbSNP), were detected and are listed as follows: rs143825456 (NM_001625.3:c.631G>A) was detected in two individuals, with one individual located in Bangkok and the other in Cape Town; rs148421308 (NM_001625.3:c.460G>A) was detected in one individual in New York City; rs61750965 (NM_001625.3:c.386G>A) was detected in one individual in New York City; and rs12116440 (NM_001625.3:c.625G>A) was detected in one individual in New York City.

**Table 2:** Previously unreported *AK2* missense variants detected in clinical trial participants located in Bangkok, Thailand, Cape Town, South Africa, and New York City, USA.

Geographic Location	Variant (ref.>alt.)	cDNA Position	Coding DNA Sequence Position	Protein Position	Amino Acid Substitution (ref.>alt.)	SIFT Prediction	PolyPhen Prediction
Bangkok	T>C	699	616	206	I>V	tolerated(0.38)	benign(0.063)
Cape Town	G>A	106	23	8	A>V	tolerated(0.27)	benign(0.002)
Cape Town	T>C	166	83	28	K>R	deleterious(0)	possibly_damaging(0.693)
Cape Town	G>A	247	164	55	A>V	deleterious(0)	possibly_damaging(0.774)
Cape Town	C>T	258	175	59	E>K	tolerated(0.29)	benign(0.01)
Cape Town	C>T	312	229	77	E>K	deleterious(0.01)	possibly_damaging(0.656)
New York City	A>C	139	56	19	V>G	deleterious(0)	possibly_damaging(0.882)
New York City	C>T	237	154	52	A>T	deleterious(0.01)	possibly_damaging(0.859)
New York City	T>C	267	184	62	K>E	tolerated(0.59)	benign(0.134)
New York City	C>T	546	463	155	E>K	tolerated(0.59)	benign(0.187)
New York City	G>A	664	581	194	T>I	deleterious(0)	probably_damaging(0.997)
New York City	T>C	699	616	206	I>V	tolerated(0.38)	benign(0.063)
New York City	T>A	699	616	206	I>F	deleterious(0.03)	benign(0.288)

Twelve previously unreported genetic missense variants were detected in 11 individuals out of 505 individuals sequenced for the coding DNA reference sequence NM_001625.3. All individuals that had detectable variants were heterozygous for those variants. One individual from Cape Town had two detectable missense variants and one individual from New York City had two detectable missense variants. Across the three geographic locations where individuals were sequenced for *AK2*, one *AK2* variant was found in both Bangkok and New York City but not in Cape Town.

### Detection of *CKM* sequence variants

For the DNA reference sequence corresponding to *CKM* (NM_001824.4), 34 total variants comprised of SNVs and deletions that are predicted to be reflected in the amino acid sequence were detected across 30 individuals. The total observed variants, including previously reported and previously unreported variants, are listed in Table B in S1 File. Twenty-nine individuals were observed to be heterozygous for all the variants detected, while one individual in New York City was observed to be homozygous. Of the 30 individuals observed to have detectable SNVs or deletions, nine individuals were located at the Bangkok study site, 12 individuals were located at the Cape Town study site, and nine individuals were located at the New York City study site. We observed two clinical trial participants exhibiting more than one variant for this kinase. One individual at the Bangkok study site was observed to carry two different variants, and one individual at the Cape Town study site was observed to carry two variants. Twenty-six previously unreported single nucleotide variants or base deletions were detected across 24 individuals (Table 3). Of the 505 individuals sequenced for *CKM* missense variants, nine individuals were observed to have a predicted “deleterious” and “probably damaging” variant, yielding a frequency of 1.8%. *In silico* tools SIFT and PolyPhen were used to predict the functional consequences of these variants. Five reported variants, found in the dbSNP, were detected: rs149354459 (NM_001824.4:c.752G>A) in one individual from Bangkok, rs17875625 (NM_001824.4:c.728G>T) in one individual from Bangkok; rs201048164 (NM_001824.4:c.163G>A) in one individual from Cape Town; rs17850202 (NM_001824.4:c.673T>C) in one individual from New York City, and rs11559024 (NM_001824.4:c.248A>G) in one individual from New York City. Of note, the individual carrying the rs11559024 variant was homozygous and, using both of the informatics tools employed this variant, was predicted to result in a decrease or loss of kinase activity.

**Table 3:** Previously unreported *CKM* missense variants detected in clinical trial participants located in Bangkok, Thailand, Cape Town, South Africa, and New York City, USA.

Geographic Location	Variant (ref.>alt.)	cDNA Position	Coding DNA Sequence Position	Protein Position	Amino Acid Substitution (ref.>alt.)	SIFT Prediction	PolyPhen Prediction
Bangkok	C>T	303	128	43	R>Q	tolerated(0.07)	benign(0.288)
Bangkok	T>C	693	518	173	Y>C	deleterious(0)	probably_damaging(0.991)
Bangkok	T>A	696	521	174	Y>F	tolerated(0.18)	possibly_damaging(0.717)
Bangkok	T> C	713	538	180	T>A	deleterious(0)	benign(0.086)
Bangkok	C> A	977	802	268	G>C	deleterious(0)	possibly_damaging(0.822)
Bangkok	T> G	1262	1087	363	M>L	tolerated(0.35)	benign(0.08)
Cape Town	G> C	451	276	92	I>M	deleterious(0)	probably_damaging(0.951)
Cape Town	G> A	569	394	132	R>C	deleterious(0)	probably_damaging(0.999)
Cape Town	C> T	623	448	150	E>K	deleterious(0.01)	possibly_damaging(0.586)
Cape Town	C> T	638	463	155	E>K	deleterious(0)	benign(0.177)
Cape Town	T> C	968	793	265	K>E	deleterious(0.04)	possibly_damaging(0.568)
Cape Town	A> G	990	815	272	M>T	deleterious(0.05)	possibly_damaging(0.503)
Cape Town	C> A	991	816	272	M>I	tolerated(0.05)	benign(0.142)
Cape Town	T> A	995	820	274	N>Y	deleterious(0)	probably_damaging(0.91)
Cape Town	C> A	1013	838	280	V>L	tolerated(0.18)	benign(0.159)
Cape Town	T> A	1032	857	286	N>I	deleterious(0)	probably_damaging(1)
Cape Town	C> G	1064	889	297	V>L	tolerated(0.21)	benign(0.299)
Cape Town	G> T	1124	949	317	L>M	deleterious(0.02)	probably_damaging(0.998)
New York City	C> A	392	217	73	G>C	deleterious(0)	probably_damaging(0.997)
New York City	T> A	435	260	87	E>V	deleterious(0.02)	benign(0.402)
New York City	T> A	482	307	103	T>S	tolerated(0.62)	benign(0.001)
New York City	T> C	720	545	182	K>R	tolerated(0.11)	benign(0.017)
New York City	A> T	806	631	211	W>R	deleterious(0)	probably_damaging(0.994)
New York City	T> G	837	662	221	D>A	tolerated(0.08)	benign(0.148)
New York City	A> G	924	749	250	F>S	deleterious(0)	probably_damaging(1)
New York City	C> T	1172	997	333	V>I	tolerated(0.3)	benign(0.085)

Twenty-six previously unreported genetic missense variants were detected in 24 individuals out of 505 individuals sequenced for the coding DNA reference sequence NM_001824.4. All individuals that had detectable missense variants were heterozygous for those variants. One participant from Bangkok had two missense variants and one participant from Cape Town had two missense variants. Across the three geographic locations evaluated for genetic variants for this kinase, no *CKM* variants were found in more than one geographic region.

### *PKM* genetic variants

For the DNA reference sequence corresponding to *PKM* (NM_001206796.1), 39 total variants comprised of SNVs and deletions that are predicted to be reflected in the amino acid sequence were detected across 33 individuals. The 39 observed variants, which include both previously reported and previously unreported variants, are listed in Table C in S1 File. All 33 individuals carrying variants in *PKM* were observed to be heterozygous for all the described *PKM* variants. Of the 33 individuals observed to have detectable SNVs or deletions, 11 individuals were located at the Bangkok study site, 10 individuals were located at the Cape Town study site, and 12 individuals were located at the New York City study site. One individual at the Cape Town study site was observed to carry two variants. Two individuals at the New York City study site were observed to carry three variants. Thirty-three previously unreported single nucleotide variations or base deletions were detected across 29 individuals (Table 4). Three reported variants, found in the dbSNP, were detected and are listed as follows: rs141732747 (NM_001206796.1:c.827C>A) was detected in one individual from New York City; rs185164430 (NM_001206796.1:c.1695C>G) was detected in one individual from New York City and rs778625515 (NM_001206797.1:c.132C>G) was detected in one individual from Cape Town. For the variants detected in *PKM*, functional predictions at the protein level were unavailable because the *in silico* tools, SIFT and Polyphen, were unable to achieve sufficient sequence diversity information in the multiple alignments necessary for prediction.

**Table 4:** Previously unreported *PKM* missense variants detected in clinical trial participants.

Geographic Location	Variant (ref.>alt.)	cDNA Position	Coding DNA Sequence Position	Protein Position	Amino Acid Substitution (ref.>alt.)	SIFT Prediction	PolyPhen Prediction
Bangkok	A>G	766	367	123	C>R	N/A	N/A
Bangkok	T>C	904	505	169	T>A	N/A	N/A
Bangkok	G>A	1007	608	203	T>I	N/A	N/A
Bangkok	T>C	1058	659	220	N>S	N/A	N/A
Bangkok	C>G	1399	1000	334	E>Q	N/A	N/A
Bangkok	C>T	1502	1103	368	R>H	N/A	N/A
Bangkok	G>A	1667	1268	423	A>V	N/A	N/A
Bangkok	C>T	1752	1353	451	M>I	N/A	N/A
Bangkok	T>C	1837	1438	480	T>A	N/A	N/A
Bangkok	A>G	2065	1666	556	W>R	N/A	N/A
Cape Town	G>A	437	38	13	T>M	N/A	N/A
Cape Town	G>C	617	218	73	A>G	N/A	N/A
Cape Town	C>T	688	289	97	A>T	N/A	N/A
Cape Town	A>T	850	451	151	S>T	N/A	N/A
Cape Town	T>C	1027	628	210	K>E	N/A	N/A
Cape Town	T>C	1412	1013	338	N>S	N/A	N/A
Cape Town	T>C	1762	1363	455	I>V	N/A	N/A
Cape Town	G>A	1792	1393	465	H>Y	N/A	N/A
Cape Town	C>T	2122	1723	575	G>S	N/A	N/A
Cape Town	G>T	2177	1778	593	S>Y	N/A	N/A
New York City	G>A	430	31	11	L>F	N/A	N/A
New York City	C>T	1345	946	316	A>T	N/A	N/A
New York City	T>C	1475	1076	359	E>G	N/A	N/A
New York City	T>A	1492	1093	365	M>L	N/A	N/A
New York City	T>C	1618	1219	407	S>G	N/A	N/A
New York City	C>T	1655	1256	419	G>D	N/A	N/A
New York City	T>A	1712	1313	438	E>V	N/A	N/A
New York City	C>A	1741	1342	448	A>S	N/A	N/A
New York City	A>T	1844	1445	482	L>H	N/A	N/A
New York City	C>T	1849	1450	484	E>K	N/A	N/A
New York City	A>G	1979	1580	527	V>A	N/A	N/A
New York City	C>T	2047	1648	550	D>N	N/A	N/A

Thirty-three previously unreported genetic missense variants were detected in 29 individuals out of 505 individuals sequenced for the coding DNA reference sequence NM_001206796.1. All individuals that had detectable variants were heterozygous for those variants. Across the three geographic locations evaluated for genetic variants for this kinase, no *PKM* variants were found in more than one geographic region. Using *in silico* tools PolyPhen and SIFT, no functional predictions were available for *PKM*. Thus, not applicable (N/A) is indicated in the table.

### *PKLR* sequence variants

For the DNA reference sequence corresponding to *PKLR* (NP_000289.1), 39 total variants comprised of SNVs and deletions that are predicted to be reflected in the amino acid sequence were detected across 35 individuals. These observed variants are listed in Table D in S1 File. Thirty-four individuals were determined to be heterozygous for the variants detected, with one individual identified as homozygous for one variant. Of the 34 individuals observed to have detectable SNVs or deletions, 10 individuals were located at the Bangkok study site, 11 individuals were located at the Cape Town study site, and 14 individuals were located at the New York City study site. Two individuals at the Cape Town study site were observed to be carrying two variants compared to four individuals at the New York City study site that carried two variants. Thirty-one previously unreported SNV or base deletions were detected across 26 individuals (Table 5). One individual from Cape Town was homozygous for the variant G180V. All other participants that showed detected missense variants were heterozygous for the variants. One individual from Cape Town carried two missense variants and four individuals from New York City had two missense variants. Across the three geographic locations evaluated for genetic variants in this kinase, no PKLR variants were found in more than one geographic region; in addition, only two of the same variants were detected in more than one individual. Of the 505 individuals sequenced for PKLR missense variants, nine individuals were observed to have at least one predicted “deleterious” and “probably damaging” variant, yielding a frequency of 1.8%. *In silico* tools SIFT and PolyPhen were used to predict the functional consequences of these variants. Six reported variants, found in the dbSNP, were detected and are listed as follows: rs147689373 (NM_000298.5:c.829G>A) was found in two individuals from New York City; rs116100695 (NM_000298.5:c.1456C>T) was found in one individual from New York City and one individual from Cape Town; rs189360283 (NM_000298.5:c.1345C>T) was found in one individual from Cape Town; rs201979697 (NM_000298.5:c.907C>T) was found in one individual from Cape Town; rs201406712 (NM_000298.5:c.1435C>T) was found in one individual from New York City and rs150077703 (NM_000298.5:c.92C>A) was found in one individual from Bangkok.

**Table 5:** Previously unreported *PKLR* missense variants detected in clinical trial participants located in Thailand, South Africa, and the USA.

Geographic Location	Variant (ref.>alt.)	cDNA Position	Coding DNA Sequence Position	Protein Position	Amino Acid Substitution (ref.>alt.)	SIFT Prediction	PolyPhen Prediction
Bangkok	G>A	89	50	17	S>F	deleterious(0)	benign(0)
Bangkok	A>T	106	67	23	L>I	tolerated(0.06)	benign(0.024)
Bangkok	C>T	161	122	41	R>Q	deleterious(0.01)	benign(0.175)
Bangkok	G>C	302	263	88	T>S	tolerated(0.16)	possibly_damaging(0.793)
Bangkok	G>C	368	329	110	A>G	deleterious(0)	possibly_damaging(0.656)
Bangkok	A>T	1240	1201	401	C>S	deleterious(0.02)	possibly_damaging(0.837)
Bangkok	A>G	1334	1295	432	V>A	deleterious(0)	benign(0.062)
Bangkok	T>C	1652	1613	538	E>G	tolerated(0.11)	benign(0.024)
Cape Town	C>T	375	336	112	M>I	deleterious(0.01)	probably_damaging(1)
Cape Town	C>A	578	539	180	G>V	deleterious(0)	probably_damaging(0.996)
Cape Town	G>T	947	908	303	P>Q	deleterious(0.05)	benign(0.13)
Cape Town	C>T	1033	994	332	G>S	deleterious(0.01)	probably_damaging(0.995)
Cape Town	A>G	1040	1001	334	M>T	deleterious(0.01)	probably_damaging(0.995)
Cape Town	A>T	1091	1052	351	L>Q	deleterious(0)	probably_damaging(0.992)
Cape Town	G>A	1313	1274	425	A>V	deleterious(0.01)	benign(0.111)
Cape Town	C>T	1343	1304	435	R>Q	tolerated(0.63)	benign(0.01)
Cape Town	T>A	1465	1426	476	T>S	tolerated(0.23)	benign(0.002)
Cape Town	A>G	1576	1537	513	F>L	tolerated(0.25)	possibly_damaging(0.624)
New York City	G>C	197	158	53	T>S	tolerated(0.47)	benign(0.008)
New York City	G>T	368	329	110	A>D	deleterious(0)	possibly_damaging(0.9)
New York City	A>G	460	421	141	F>L	tolerated(0.45)	benign(0.082)
New York City	G>A	473	434	145	P>L	tolerated(0.1)	possibly_damaging(0.653)
New York City	A>T	524	485	162	I>N	deleterious(0)	probably_damaging(0.999)
New York City	G>A	530	491	164	T>I	deleterious(0.01)	probably_damaging(1)
New York City	A>G	580	541	181	S>P	tolerated(0.26)	benign(0.315)
New York City	A>T	866	827	276	V>E	deleterious(0)	probably_damaging(0.986)
New York City	A>C	1230	1191	397	D>E	deleterious(0.01)	possibly_damaging(0.872)
New York City	C>T	1234	1195	399	A>T	tolerated(0.29)	probably_damaging(0.94)
New York City	G>A	1448	1409	470	A>V	deleterious(0.02)	probably_damaging(0.995)
New York City	A>G	1520	1481	494	I>T	deleterious(0)	probably_damaging(0.985)
New York City	A>G	1583	1544	515	L>S	deleterious(0.03)	probably_damaging(0.97)

Thirty-one previously unreported genetic missense variants were detected in 26 individuals out of 505 individuals sequenced for the coding DNA reference sequence NP_000289.1.

### In vitro phosphorylation of tenofovir and tenofovir-monophosphate by AK2 and AK2 variants that were detected in study participants

**Fig 2:**
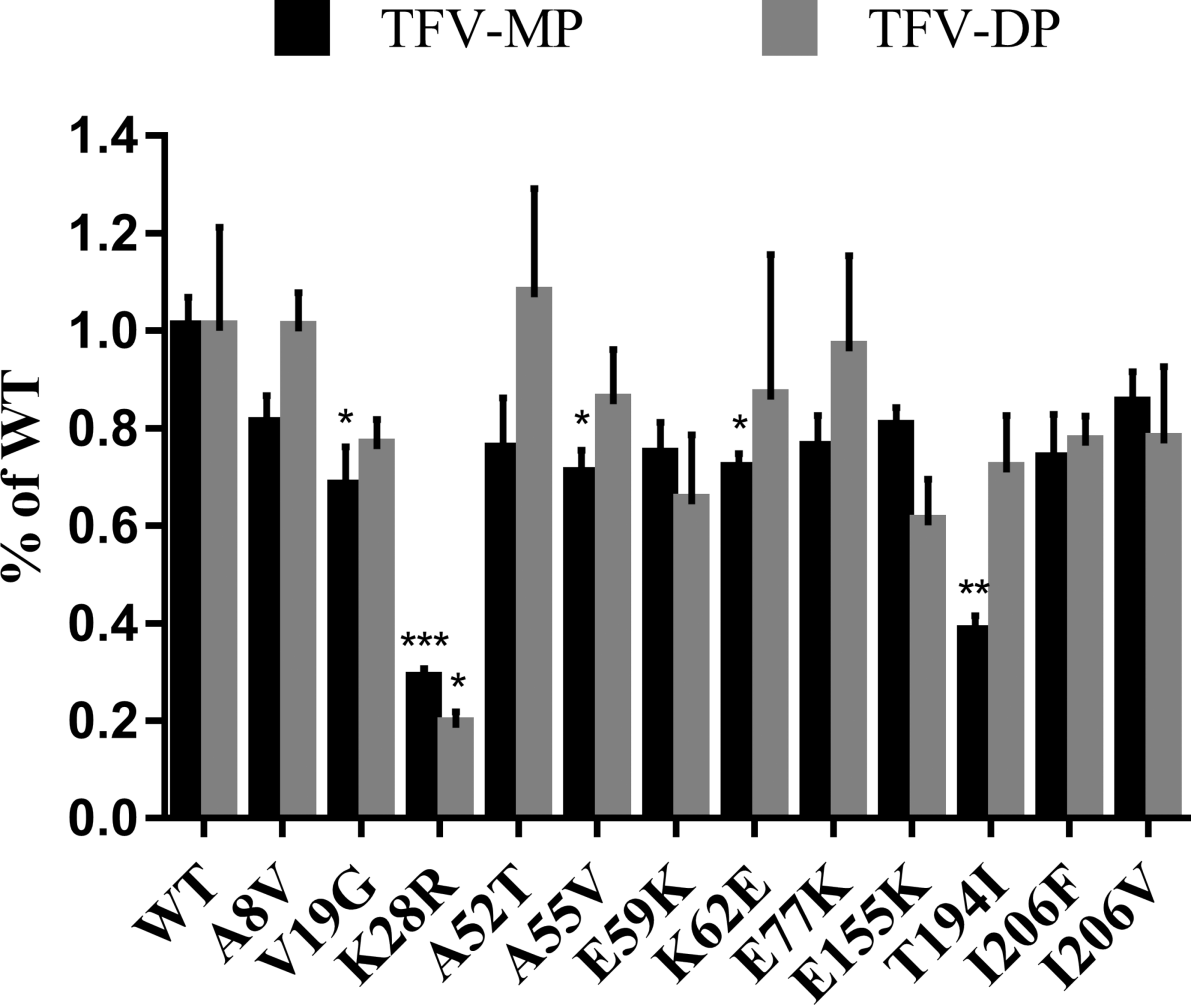
Naturally occurring genetic variants of AK2 and the resulting impact on TFV phosphorylation. Wild-type (WT) AK2 and variants were expressed in and purified from *E*. *coli* in order to evaluate their effects on TFV phosphorylation in vitro. Proteins were incubated with 1 mM TFV in assay buffer for 2.5 h before reaction was quenched. A saturating concentration of TFV was used in order to ensure that substrate depletion would not play a causal role in differences observed in the formation of phosphates in these activity assays. TFV-MP and TFV-DP were detected by uHPLC-MS/MS where signal to noise ratio was used to establish the corresponding bar graph. Error bars represent standard deviation; n = 3. A two-tailed unpaired *t* test and significance was set as follows: *, p≤0.05; **, p≤0.01; ***, p≤0.001.

Since AK2 plays a critical role in TFV activation through carrying out the first phosphorylation step that results in the formation of TFV-MP, we used AK2 activity assays as a model system for testing the predictions regarding the impact of genetic variants on activity towards TFV. AK2, as well as the 12 variants that were identified in study participants, were expressed and purified in E. coli followed by the performance of assays to measure the ability of these enzymes to phosphorylate TFV. To do this, we established an AK2 activity assay for TFV as well as an uHPLC-MS/MS that enabled the direct detection of TFV metabolites. We found that AK2 not only phosphorylated TFV to TFV-MP, but further catalyzed the phosphorylation of TFV-MP to TFV-DP (Fig 2). In addition, several of the variants exhibited differential activity towards TFV as compared to the wild-type enzyme. Of the 12 variants we tested, V19G, K28R, A55V, K62E, and T194I variants were found to be functionally affected, leading to statistically significant decreases in the formation of TFV-MP to 67.4 ± 8.8%, 28.0 ± 2.8%, 70.0 ± 5.6%, 70.1 ± 3.9%, and 37.5 ± 4.0%, respectively, of that formed by the wild-type enzyme. Of note, the K28R variant also exhibited a marked decrease in the formation of TFV-DP to 18.7 ± 3.2% compared to wild-type. Interestingly, of the 5 abovementioned variants that exhibited decreased kinase activity toward TFV in our in vitro assays, 4 had been predicted using the SIFT and PolyPhen bioinformatics tools to have decreased activity compared to wild-type.

## Discussion

This study identified 103 previously unreported genetic variants of the kinases *AK2*, *CKM*, *PKM*, and *PKLR* in 505 clinical trial participants located in Thailand, Cape Town, and the United States. Using the SIFT and PolyPhen bioinformatics tools it was predicted that 19 of the 505 individuals carried variants that would result in a decrease or loss of enzymatic activity. The breakdown for this was one individual with an AK2 variant predicted to be deleterious or possibly damaging and 9 individuals each with CKM or PKLR variants predicted to negatively impact kinase activity. Each variant in this study was unique, in that it was only detected in one individual. While next-generation sequencing has been applied to genomic DNA extracted from dried blood spots in prior studies [20–23], interrogating this extracted DNA for sequence variants in these kinases has not yet been carried out. To increase confidence in the variants identified, our analyses conformed to the American College of Medical Genetics and Genomics clinical laboratory standards for next-generation sequencing [24]. To compare frequencies in this study of previously reported genetic variants, data from the 1000 Genomes Project [25] and the Exome Aggregation Consortium (ExAC) [26], which includes over 60,000 genotyped, unrelated individuals, was used because of the population and, in the case of the 1000 Genomes Project, location information these datasets provide.

In comparison to a previous analysis from our laboratory that sequenced 142 women from the geographic regions Durban, South Africa, Kampala, Uganda, and four locations within the United States [9], we detected one overlapping variant for the four kinases sequenced. Of note, in the abovementioned study, there was no overlap in the variants that were detected among the participants. With this in mind it is interesting that *PKM* SNP rs778625515 (NM_001206796.1:c.354C>G) was detected in one clinical trial participant at the USA study site from our previous analysis and shared with one participant at the Cape Town study site in our current study. At the protein level, this *PKM* variant is predicted to result in a mutation from asparagine to lysine at amino acid residue 118 (N118K). This mutation is located adjacent to a beta strand comprised of amino acids 119 to 121 [27]. Because of its location two amino acids away from a reported ATP binding site, it is possible binding of this substrate will be disrupted, affecting kinase function; however, as noted above, the gene encoding *PKM* currently has no functional prediction of this variant using *in silico* tools PolyPhen or SIFT. Functional predictions of the variants detected in *PKM* at the protein level were unavailable because the *in silico* tools used for prediction, SIFT and PolyPhen, were not supplied with sufficient sequence diversity required for the performed multiple sequence alignments necessary for the algorithms used in these predictions.

In the present study, we detected the SNV rs147689373 (NM_000298.5:c.829G>A) of *PKLR* in two individuals in New York City, yielding a 1.3% (2/149) frequency within New York City and a 0.40% (2/505) frequency in our study. Of note, this variant was detected in one participant in South Africa in our previous study [9]. In the 1000 Genomes Project, the overall frequency for this variant was 0.26%. The only location in the USA included in the 1000 Genomes Project reporting frequency was 0.78% in the Mexican Ancestry from Los Angeles USA. The frequency of this variant in the 1000 Genomes Project from other locations was 2.1% in African Caribbeans in Barbados, 1.0% Esan in Nigeria, 1.52% Luhya in Webuya Kenya, 1.18% Mende in Sierra Leone, and 0.46% Yoruba in Ibadan, Nigeria. At the protein level, this variant results in a mutation at the 277^th^ amino acid with a substitution from glutamic acid to lysine. Other previously reported variants around this region of the protein have been associated with pyruvate kinase deficiency [28].

Of the previously reported variants detected in our study, the SNP in *CKM* rs11559024 (NM_001824.4:c.248A>G) was the only variant reported at an overall frequency greater than 1% across the ExAC database. This variant was detected in one individual from New York City, exhibiting a 0.20% (1/505) overall frequency across the three geographic locations in this study and a 0.67% (1/149) frequency in the New York City population we examined. Across data listed in the ExAC database, a total frequency of 1.1% throughout all the subpopulations included in the database is reported. All subpopulations exhibited frequencies of this variant, with Finnish European at 1.94%, non-Finnish European at 1.51%, South Asian at 0.58%, Latino at 0.36%, African at 0.36%, and the East Asian population exhibiting 0.01% frequency. Consulting the 1000 Genomes Project, this variant is reported at the following frequencies in the following sites located in the USA: 0.25% in Utah Residents with Northern and Western European Ancestry, 0.49% in Gujarati Indians from Houston, Texas population, and 0.78% in individuals reporting Mexican Ancestry from Los Angeles USA. Populations from the 1000 Genomes Project outside the United States showed frequencies at 0.58% in Bengali from Bangladesh, 0.49% in Han Chinese in Beijing, 0.53% in Colombians from Medellin, Colombia, 1.52% in Finnish in Finland, 1.65% in British in England and Scotland, 1.04% in Punjabi from Lahore, Pakistan, and 0.48% in Puerto Ricans from Puerto Rico. Overall, in the populations included in the 1000 Genomes Project combined, the frequency of this variant was 0.38%—a higher overall frequency than that observed in our study. The resulting protein from this variant would exhibit a residue change at the 83^rd^ amino acid of the protein, resulting in the mutation of a glutamic acid to glycine. *In silico* functional prediction tools, predict that this substitution would be “deleterious” using the SIFT algorithm and “possibly damaging” using PolyPhen.

To test these *in silico* predictions, AK2 and the 12 AK2 genetic variants that were identified in this study were generated in vitro to examine their activity towards TFV. To confirm that our assay was viable, we first looked at the activity of WT-AK2, previously suggested to initiate the first phosphorylation step of TFV [9], towards TFV. We found that AK2 indeed phosphorylated TFV to TFV-MP; however, interestingly we also found that AK2 could catalyze the phosphorylation of TFV-MP to TFV-DP. Utilizing the same assay, we investigated the 12 variants and their effect on activity towards TFV. The V19G, K28R, A55V, K62E, and T194I variants were found to negatively impact kinase activity, leading to a decrease in the formation of TFV-MP compared to wild-type;however, only K28R and T194I exhibited a marked decrease in TFV phosphorylation in our functional assays. Future studies using a range of TFV concentrations in these assays are required to gain a more complete understanding of the impact of AK2 genetic variants on phosphorylation since the use of a single TFV concentration, as was done in the present study, may not fully reveal the effects of these AK2 variants on activity. Interestingly, in line with our finding that AK2 can also phosphorylate TFV-MP to TFV-DP, the K28R exhibited a decrease in the formation of TFV-DP compared to wild-type. Further, since the participants in our study were all heterozygous for these variants, it is difficult to predict from our data what the clinical impact might be; however, given the possibility that there are individuals homozygous for these variants our functional data shed light on the potential for differences in TFV activation based on genotype. To this end, the K28R variant is of particular interest since clinically, it has been demonstrated that a genetic mutation at this same residue is associated with a loss of AK2 activity (AK2 K28E variant) that results in reticular dysgenesis and disruptions in normal cellular function [11, 12]. The lysine at position 28 is reported to be in the middle of the ATP binding domain of AK2 [27], the region that plays a role in catalyzing the transfer of a phosphate group from an ATP molecule and mutations within the ATP binding domain could functionally affect this activity. The lysine to arginine mutation may be disrupting the source of phosphate to transfer onto TFV, which would explain the decrease in both TFV-MP and TFV-DP formation. The decrease in TFV-MP formation seen in the other variants suggests that these amino acid residues may be important in the binding of TFV. However, since we demonstrated that AK2 also has activity towards TFV-MP that results in the formation of TFV-DP, we cannot rule out the possibility that the decrease in TFV-MP formation is due to increased TFV-DP formation. To elucidate this, future studies to investigate the enzyme kinetics of the phosphorylation of TFV and TFV-MP are required, in addition to biophysical studies to probe the binding of TFV to the AK2 variants.

Our observation of little overlap between individuals in the variants identified suggests that while genetic differences in these kinases exist, the variants are of low frequency. This makes the probability of detecting mutations that might affect TFV activation in a given population difficult to ascertain. Further, since several enzymes are involved in the cascade of TFV activation, the actual clinical impact of a variant of one enzyme is difficult to predict and it is likely that for substantial loss in TFV activation to occur an individual would need to carry variants of more than one enzyme. In addition, due to the low frequency of the genetic variants detected within the HPTN 067 study participants, correlations between TFV and TFV-DP levels and our data were not possible. Further, adherence is an issue that impacts HIV PrEP studies and this makes it difficult to elucidate the impact of genotype on study drug levels when the number of individuals carrying genetic variants is low. Thus, in order to perform a powerful analysis, a future study is required in which directly observed therapy is employed and participants are enrolled based on genotype, similar to a previous study that we have performed investigating the impact of genetics on the metabolism of the anti-HIV drug maraviroc [29]. Importantly, our results demonstrate that the extraction of genomic DNA from dried blood spots can facilitate the detection of tenofovir-activating kinases genetic variants, indicating that this low-cost, limited sample method can be used in large-scale clinical trials for tenofovir pharmacogenetics applications. In addition, these data offer new, previously unreported variants across the *AK2*, *CKM*, *PKM*, and *PKLR* genes, which encode the kinases that play a role in tenofovir activation. Of note, several of these kinases were predicted to result in a loss or a decrease in the function of the protein and our functional assays have provided verification that naturally occurring genetic variants can have an effect on TFV phosphorylation. Taken together, this work suggests that divergence in drug concentrations observed among PrEP users could be due to genetic variation in TFV activation rather than adherence alone.

## Supporting information

S1 File (**Table A**). Detailed genotyping data for AK2 (**Table B**). CKM (**Table C**). PKM (**Table D**). PKLR.
(XLSX)
